# Autoinducer 2 as a universal language in microbial consortia: decoding molecular mechanisms, ecological impacts, and application

**DOI:** 10.1080/19490976.2026.2615494

**Published:** 2026-01-23

**Authors:** Shuyu Guo, Bingyong Mao, Xin Tang, Qiuxiang Zhang, Jianxin Zhao, Wei Chen, Shumao Cui

**Affiliations:** a State Key Laboratory of Food Science and Resources, Jiangnan University, Wuxi, People's Republic of China; b School of Food Science and Technology, Jiangnan University, Wuxi, People's Republic of China; c MOE Medical Basic Research Innovation Center for Gut Microbiota and Chronic Diseases, School of Medicine, Jiangnan University, Wuxi, Jiangsu, People's Republic of China; d National Engineering Research Center for Functional Food, Jiangnan University, Wuxi, Jiangsu, People's Republic of China; e International Joint Research Laboratory for Maternal-Infant Microbiota and Health, Jiangnan University, Wuxi, Jiangsu, People's Republic of China

**Keywords:** Autoinducer 2, quorum sensing, synthesis, multi-receptors, interspecific interactions, application, regulation, production

## Abstract

In natural and engineered ecosystems, diverse species interact in complex ways to form highly efficient microecologies. One key orchestrator of these interactions is autoinducer-2 (AI-2), a signaling molecule that plays a crucial role in microbial community assembly, metabolic flux, and resilience to environmental disturbances. This review provides the systematic synthesis of AI-2’s dual structural dynamics (S-THMF-borate/R-THMF interconversion) and its context-dependent roles in mediating bacterial crosstalk. It also reveals the receptor diversity (such as LuxP and LsrB) of AI-2 in bacterial kingdom and the signal transduction mechanism. Systematically elaborated on AI-2’s regulation of cellular metabolic flux and its ability to autonomously exhibit a series of coordinated behaviors in response to environmental changes. The review explores the ramifications of AI-2 on bacterial community interactions in synthetic biology and natural ecosystems. The wide application of AI-2-mediated interspecific communication in various fields including host health, agriculture, industry and environmental ecology has also been widely discussed. Factors influencing AI-2 production are thoroughly examined, including internal factors such as strain specificity, cell density, growth form and the phenotypic heterogeneity. Additionally, external biological factors (such as nutritional status and environmental stress) and abiotic factors (aggregation, diffusion, and flow) are discussed in detail. By examining knowledge gaps in AI-2-mediated spatial heterogeneity and multi-QS system coordination, this work charts a roadmap for harnessing microbial communication in chemical engineering and environmental sustainability.

## Graphical abstract

**Figure:**
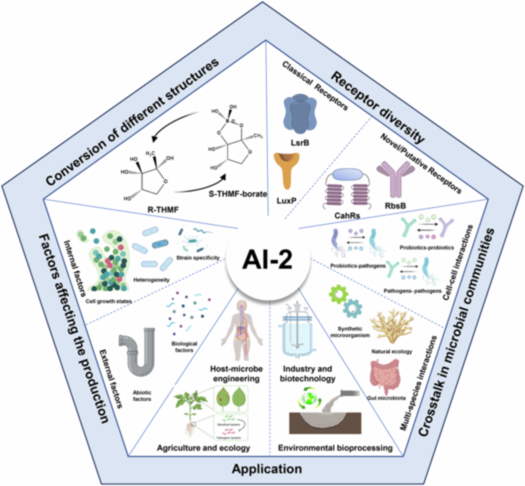

## 1. Introduction

In natural environments (including intestinal microecology), microorganisms often coexist with diverse species. The inherent adaptability of bacteria across various environments stems from their remarkable ability to sense and respond to fleeting environmental changes.^1^ This capability enables them to thrive under different conditions, positioning them as pivotal players in ecological systems.^2^ While species-specific recognition allows bacteria to identify themselves within mixed populations, they often rely on one or more mechanisms to detect the presence of other species.^3^ They can dynamically adjust gene expression and behavior by monitoring the ratio of their own species to others. Microbial communities thrive through intricate interactions that dictate ecological stability and functional versatility. Consequently, interspecific communication is crucial for their survival and proliferation.

Microorganisms frequently inhabit complex, rapidly changing environments.^4^ To adapt effectively, bacteria have evolved sophisticated strategies, such as quorum sensing (QS).^4^ This mechanism involves the production, detection, and response to extracellular signaling molecules called autoinducers. By enabling mutual perception, QS modulates gene expression related to various cellular processes, coordinates multicellular behavior, and is essential for bacterial adaptation and survival.^4^ Recent research has focused on the evolution of QS types in dynamic habitats and the communication it facilitates for both intraspecific and interspecific cooperation or competition.^5‐7^

AI-2 is the only known interspecies signaling molecule found in both Gram-positive and Gram-negative bacteria, making it a widely perceived and responsive molecule within diverse bacterial communities.^8^ However, not all bacteria that produce AI-2 either have sensors or trigger a response to AI-2.^9^ Some scholars believe that AI-2 can sometimes be a signal, a cue or metabolic by-product used opportunistically for signaling depending on the bacterial producer or receiver.^9‐11^ In other words, the signaling function of AI-2 might be the result of a co-opted consequence of its metabolic ubiquity. At present, the proposal of AI-2 as a key interspecies signal in the microbiome has been widely recognized.^12^ AI-2 has emerged as a linchpin in deciphering bacterial interactions, transcending its classical role in QS to mediate metabolic cooperation, competition, and host-microbe dialogs.^4^^,^^13^ While its synthesis is generally consistent across bacterial types, significant differences exist in its signaling and sensing mechanisms. While previous studies focused on AI-2-driven synchronized behaviors, understanding of its synthesis process, production regulation, and role in enabling context-specific communication across diverse niches remains limited.

This article offers a comprehensive overview of the biosynthetic pathways and receptors related to AI-2. It also delves into the discovery of AI-2 as an interspecific signaling molecule and its effects on interactions between and among bacterial species. The article examines various internal and external factors influencing AI-2 production and presents practical applications of interspecific communication mediated by AI-2. Finally, it highlights gaps in current research and outlines key issues that need to be addressed in future studies on this topic. This review synthesizes mechanistic breakthroughs and translational applications of AI-2, offering a roadmap for leveraging microbial crosstalk in biotechnology.

## 2. The synthesis of AI-2 involved in activated methionine cycle (AMC)

The structural plasticity of AI-2, manifested through S-THMF-borate and R-THMF interconversion, has been identified as the molecular basis for environment-dependent interspecies signaling. AI-2 biosynthesis is tightly coupled to the AMC, forming an integrated network with acyl-homoserine lactones (AHLs) and cholera autoinducer-1 (CAI-1) systems to hierarchically regulate microbial behaviors across taxonomic boundaries. This metabolic integration may support the model in which AI-2 can act as a central regulator for cross-species behaviors.

### 2.1. Biosynthesis pathways: metabolic cross-talk with AMC

**Figure 1.**
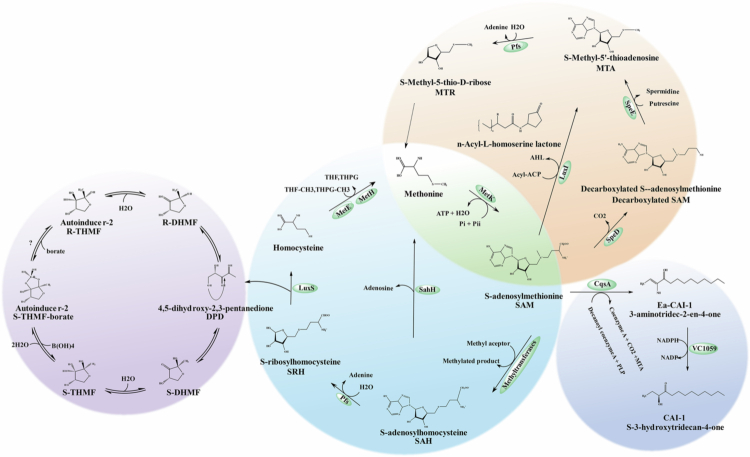
Metabolic network of AI-2 biosynthesis and cross-talk with AMC-derived signals. Pink circle: the interconversion of boronated (S-THMF-borate) and non-boronated (R-THMF) AI-2 isoforms. Blue circle: the AMC-driven precursor synthesis pathway, specifically the conversion of SAM to DPD (the core precursor of AI-2). Orange circle: the coupling of the Yang cycle with AHLs synthesis. Purple circle: biosynthetic pathway of CAI-1, another quorum-sensing molecule. This integrated map illustrates how AI-2 intersects with multiple quorum-sensing signals and central metabolic circuits, laying the groundwork for both intra- and inter-species communication. Note: AI-2: autoinducer-2; AMC: activated methionine cycle; S-THMF-borate: (2S,4S)-2-methyl-2,3,3,4-tetrahydroxytetrahydrofuryl borate; R-THMF: (2 R,4S)-2-methyl-2,3,3,4-tetrahydroxytetrahydrofuran; SAM: S-adenosylmethionine; DPD: 4,5-dihydroxy-2,3-pentanedione; AHLs: acyl-homoserine lactones; CAI-1: cholera autoinducer-1;S-THMF: (2S,4S)-2-methyl-2,3,3,4-tetrahydroxytetrahydrofuryl; S-DHMF: (2S,4S)-2-methyl-2,4-dihydroxytetrahydrofuran; R-DHMF: (2 R,4S)-2-methyl-2,4-dihydroxytetrahydrofuran.

AI-2 biosynthesis is tightly coupled to the AMC, with S-adenosylmethionine (SAM) functioning as the central methyl donor (Figure 1). SAM synthesis from methionine is catalyzed by methionine adenosyltransferase (MetK), and methyl-transfer to multiple molecules in the cell yields S-adenosylhomocysteine (SAH) as a byproduct of methyltransferase reactions.^14^ SAH is subsequently cleaved to form homocysteine (Hcy) and S-ribosylhomocysteine (SRH), with concomitant generation of the AI-2 precursor 4,5-dihydroxy-2,3-pentanedione (DPD).^11^ The labile DPD intermediate undergoes spontaneous cyclization, generating furan derivatives that serve as direct AI-2 precursors. Further modifications ultimately yield mature AI-2 signaling molecules.

Two biologically active AI-2 isoforms were originally characterized in *Vibrio harveyi* (*V. harveyi*) and *Salmonella enterica* serovar Typhimurium.^3^ In *V. harveyi,* AI-2 was structurally resolved as (2S,4S)-2-methyl-2,3,3,4-tetrahydroxytetrahydrofuran-borate (S-THMF-borate) through crystallographic analysis of LuxP-AI-2 complexes.^11^ S-THMF-borate biosynthesis occurs *via* borate-dependent condensation of (4S)-DPD, which triggers the bioluminescence cascade in *V. harveyi*. The *Salmonella* LsrB receptor was instrumental in identifying the boron-free AI-2 isoform, R-THMF. R-THMF arises from water-mediated cyclization of DPD, producing the (2 R,4S) stereoisomer.^15^ Then, AI-2 signaling has since been documented in *E. coli* and diverse taxa including *Rhizobiaceae*, *Bacillus* spp., and *Clostridium* spp.^16^

### 2.2. Structural dynamics of two forms of AI-2

Structural interconversion between S-THMF-borate and R-THMF isoforms has been demonstrated, a process enabled by the keto-enol tautomerism of the DPD precursor.^15^^,^^17^ The hydrophilic nature of DPD facilitates rapid equilibrium among multiple isomeric forms in aqueous environments.^18^ A borate supplementation was shown to drive the equilibrium toward S-THMF-borate formation.^15^ This environmentally regulated equilibrium provides microbial communities with a bimodal signaling capability, establishing AI-2 as the predominant interspecies communication system mediating host-microbe crosstalk, environmental resilience, and ecosystem homeostasis.^19^

As established earlier, boron is not an obligatory component of all AI-2 variants. Despite being structurally dispensable, boron critically modulates AI-2-mediated signal propagation.^11^^,^^20^ In *V. harveyi*, borate catalyzes DPD conversion to stabilized S-THMF-borate through pro-AI-2 intermediates, potentiating LuxPQ-dependent bioluminescence.^3^ Boron compounds (H_3_BO_3_, NaBO_2_, etc.) differentially regulate AI-2 activity, altering biofilm and flagellar gene expression in *E. coli.*^21^ Key knowledge gaps persist regarding boron’s AI-2 functions, encompassing: (I) taxonomic distribution of borated AI-2 production, (II) molecular mechanisms of boron-mediated signaling, and (III) obligatory requirements for boron in QS pathways.

### 2.3. AMC facilitates AI-2 as a universal language for bacteria

SAM generated through the AMC serves as the biosynthetic precursor for AHLs, which mediate intercellular communication in Gram-negative bacteria. SAM is concurrently utilized for CAI-1 autoinducer production in *Vibrio cholerae*. *Vibrio cholerae* employs two parallel QS circuits: the CAI-1/CqsS and AI-2/LuxS systems.^22^ These autoinducers converge onto a shared signal transduction cascade, coordinating virulence factor production, biofilm formation, and bioluminescence.^23^ Although CAI-1 is typically regarded as the dominant signal in *Vibrio cholerae,*^24^ exogenous AI-2 was demonstrated to elicit stronger QS responses in specific experimental conditions. This phenotypic variation may reflect strain-specific genetic backgrounds, growth phase-dependent regulation, and environmental context.^22^ Furthermore, as a facultative aerobic bacterium, the amounts of the two types of AI produced by *Vibrio cholerae* are determined by the oxygen level.^25^ Therefore, the diverse AI-2 QS system within *Vibrio cholerae* supports the response of the bacteria to various environmental, host and cell density signals.^25^^,^^26^ Besides, when *Escherichia coli* and *V. harveyi* were co-cultured, the production of AI-2 by either bacterium could regulate the light production of *V. harveyi* and trigger *lsr* induction in *Escherichia coli.*^27^ This study not only confirmed that AI-2 can mediate bidirectional communication among different bacterial species, but also indicated that AI-2 can complete cross-structural transformation and then be recognized.^27^

The AMC thus functions as a metabolic nexus, simultaneously supporting AI-2, AHLs, and CAI-1 biosynthesis to enable hierarchical control of microbial social behaviors. The coexistence of multiple QS systems permits integrated responses to biotic and abiotic stimuli, maximizing adaptive potential.^13^ Conversely, QS systems reciprocally regulate AMC flux, as evidenced by *Burkholderia cepacia*’s precise control over signaling molecule biosynthesis and cellular methylation.^28^

## 3. Sensing of AI-2 by multi-receptors

The perception of AI-2 among bacterial species is complex, mediated by a diverse set of receptors. It is critical to distinguish between receptors with direct biochemical and structural evidence for AI-2 binding and those for which the designation is primarily inferred from genetic or homology-based evidence. Currently, two receptor types, LuxP and LsrB, are definitively established as bona fide AI-2 receptors through crystallographic and binding assays. Other proteins, including several containing dCACHE domains (categorized here as CahRs), GAPES1, and FruA, have been proposed as novel AI-2 receptors, but the evidence for most is still emerging and often indirect. This section details the well-characterized systems and critically evaluates the evidence for proposed receptors.

### 3.1. LuxP

**Figure 2.**
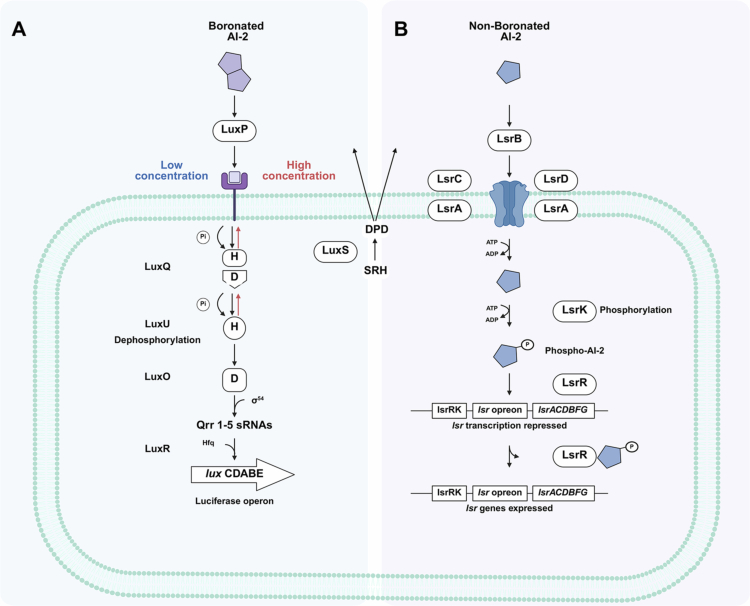
Two major AI-2 receptor paradigms determine species-specific signal recognition. (A) LuxP/LuxQ pathway for boronated AI-2 (exemplified by *Vibrio harveyi*). Boronated AI-2 binds to the periplasmic receptor LuxP, triggering a phosphorelay cascade through LuxQ, LuxU, and LuxO. This ultimately modulates the expression of quorum-sensing-regulated genes (such as the luciferase operon *lux*CDABE) *via* Qrr1-5 sRNAs and LuxR. (B) The LsrB transporter of non-boronated AI-2 (exemplified by *Bacillus cereus*, *Escherichia coli*, and *Salmonella typhimurium*). In this pathway, non-boronated AI-2 is transported into the cell by the LsrBCD transporter complex. Once inside, AI-2 is phosphorylated by LsrK, which relieves the transcriptional repression of the lsr operon by LsrR, enabling expression of *lsrACDBFG* genes involved in AI-2 processing and signaling. This figure highlights that AI-2 employs distinct receptor systems across bacterial species, with the boronation status of AI-2 acting as a key determinant of pathway specificity.

LuxP is one of the most well-defined AI-2 receptors, with crystal structures of the LuxP-AI-2 complex providing direct visual evidence of binding. As illustrated in Figure 2A, the AI-2 receptor in *V. harveyi* is LuxP, a periplasmic binding protein that interacts with the membrane-spanning sensor kinase LuxQ, forming the LuxPQ two-component system. LuxQ relays the signal to the regulatory protein LuxO through LuxU, a phosphotransferase. LuxO, a σ^54^-dependent transcription activator, promotes the expression of the luciferase structural genes (*luxCDABE*) and their regulatory inhibitors. At low cell density, boron-bound AI-2 binds to LuxP, initiating a phosphorylation cascade through LuxQ, LuxU, and LuxO.^29^ LuxQ dephosphorylation triggers a reverse phosphorelay, ultimately inhibiting LuxR. Specifically, phosphorylated LuxO interacts with σ^54^cto activate transcription of five small regulatory RNAs (Qrr1-5), which, together with Hfq, destabilize *luxR* mRNA, suppressing LuxR production.^30^ At high cell density, AI-2 binding to LuxP blocks LuxQ autophosphorylation, converting it from a kinase to a phosphatase. Dephosphorylated LuxO exhibits reduced activity, repressing *qrr* transcription. In the absence of Qrr sRNAs, *luxR* transcription is activated, increasing LuxR levels and upregulating the *lux* operon.^31^

### 3.2. LsrB

Similar to LuxP, the LsrB receptor has been rigorously characterized (Figure 2B). Structural studies have unequivocally shown that LsrB binds non-borated AI-2 in its periplasmic binding pocket. In *Salmonella typhimurium*, *Bacillus cereus*, *Clostridium saccharobutylicum* and *E. coli*, the non-borated AI-2 receptor is LsrB, a high-affinity periplasmic substrate-binding protein.^32^ AI-2 is recognized and internalized by the ABC transporter complex LsrACDB, which consists of the permease components LsrC and LsrD and the ATP-binding subunit LsrA. Following uptake, AI-2 is phosphorylated by the kinase LsrK to form phospho-AI-2,^33^ a key step in activating the QS cascade. Phospho-AI-2 subsequently binds to the transcriptional repressor LsrR, which under basal conditions inhibits the *lsr* operon.^34^ This interaction induces LsrR dissociation from the *lsr* operon, thereby derepressing its expression.^29^ The LsrACDB transporter further facilitates AI-2 uptake, creating a positive feedback loop that sustains the QS response. Contrary to the initial belief that Lsr systems were restricted to Gram-negative bacteria, homologous systems have been identified in some Gram-positive bacteria, including *Bacillus* and *Clostridium species.*^32^^,^^35^ However, current reports lack research on the functional role of this system in these organisms.

### 3.3. dCACHE domain-containing proteins

A diverse family of transmembrane proteins containing dCACHE domains has been implicated in AI-2 sensing. The evidence for direct AI-2 binding, however, varies considerably among its members. For some members, such as *Bacillus subtilis* histidine kinase KinD and *Rhodopseudomonas palustris* diguanylate cyclase rpHK1S-Z16, biochemical and structural studies have provided direct evidence for AI-2 binding to their dCACHE domains, which consequently enhances their enzymatic activity.^16^ In other cases, the evidence is more indirect. For example, in Pseudomonas aeruginosa, the chemotaxis receptors PctA and TlpQ are proposed to bind boron-free AI-2 based on genetic and behavioral assays (AI-2-induced chemotaxis).^36^ However, direct biochemical confirmation of AI-2 binding to these specific receptors is still needed. Similarly, broader bioinformatic analyzes suggest that methyl-accepting chemotaxis proteins (MCPs) containing dCache_1 domains can detect AI-2,^37^ but this designation for most MCPs is inferred from domain homology and requires individual experimental validation.

### 3.4. GAPES1

The GAmmaproteobacterial PEriplasmic Sensor 1 (GAPES1) has been proposed as a novel class of extracellular AI-2 receptors in Gram-negative bacteria.^38^ Structural analyzes suggest that the *N*-terminal GAPES1 domain binds AI-2 and possesses diguanylate cyclase (DGC) activity, potentially linking AI-2 sensing to c-di-GMP signaling.^39^ The initial identification and phylogenetic conservation of GAPES1 proteins within *Enterobacterales* are compelling, but further functional studies across different homologs are required to solidify its role as a widespread and direct AI-2 receptor.^38^

### 3.5. FruA

FruA in *Streptococcus pneumoniae* represents a highly speculative case of a putative AI-2 receptor. It was initially identified as a candidate through genetic screens.^40^ Evidence for its involvement in AI-2 signaling is entirely indirect, relying on phenotypic observations that exogenous AI-2 influences carbon metabolism and virulence in a FruA-dependent manner.^41^ Crucially, as noted in the original study and still true today, there is a lack of direct biochemical evidence (like binding assays) demonstrating that FruA physically binds AI-2. Therefore, it remains uncertain whether FruA is a genuine receptor or merely a component of a downstream pathway indirectly affected by AI-2 signaling.

## 4. AI-2 involved in quorum sensing for interspecific interactions

AI-2 is capable of regulating cellular metabolic flux and autonomously exhibiting coordinated behavior in response to environmental changes. In these biological processes, AI-2 mediates diverse cell-cell interactions, including pathogen-pathogen, pathogen-probiotic, and probiotic-probiotic interactions, as well as multi-species interactions in synthetic biology, gut microbiota, and natural ecosystems.

### 4.1. Collective coordinated behavior mediated by AI-2

#### 4.1.1. Remodeling bacterial metabolism

**Figure 3.**
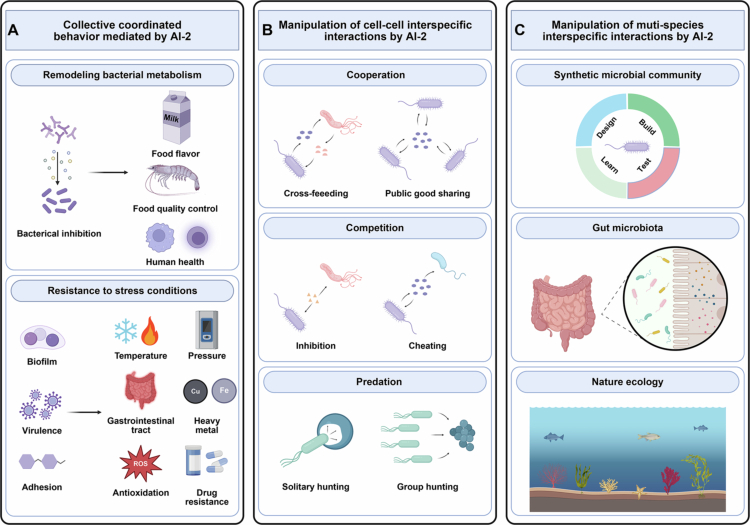
Collective coordinated behavior mediated by AI-2 and its impact on cell-cell and multi-cell interactions. (A) AI-2-coordinated collective behaviors, including remodeling of bacterial metabolism and enhancement of stress resistance. (B) Manipulation of cell-cell and interspecific interactions by AI-2. AI-2 shapes intra- and interspecies dynamics through three core mechanisms: cooperation, competition, and predation. (C) Manipulation of multi-species interspecific interactions by AI-2. AI-2 facilitates the construction and functional regulation of synthetic microbial communities, with applications in natural ecosystems and human health (such as gut microbiota modulation). The figure integrates how a shared chemical language (AI-2) coordinates complex social ecology in bacterial populations.

The AI-2 QS system plays a crucial role in regulating bacterial metabolism (Figure 3A). In *Lactobacillus plantarum* L3, phenyllactic acid production was regulated by the LuxS/AI-2 system, resulting in significant antifungal activity.^42^ In *Limosilactobacillus fermentum* A119, the AI-2 QS system had been shown to mediate the expression of aldehyde dehydrogenase, catalyzing the conversion of benzaldehyde to benzoic acid. During coculture, the transcription of the QS *luxS* gene and bacteriocin regulatory operons (plnB and plnC) in *Lactobacillus plantarum* AB had been shown to be significantly increased, resulting in effective inhibition of spoilage organisms, primarily *Shewanella baltica*, in shrimp samples.^43^ Lipolic acid activated the luxS/AI-2 QS system in *Limosilactobacillus fermentum* L1, leading to increased expression of myosin cross-reactive antigen protein, enolase, and the production of conjugated linoleic acid (CLA).^44^ Furthermore, the AI-2 QS system enhanced galactose utilization in *Streptococcus suis*, upregulated the Leloir pathway for capsular polysaccharides precursor production, and boosts capsular polysaccharides synthesis, resulting in increased resistance to macrophage phagocytosis.^45^ Utilizing QS quenching principles, a synthetic mammalian cell-based microbial-control device was developed.^46^ This device detected formyl peptides secreted from various microbes with high sensitivity and responded with robust AI-2 production, resulting in control of QS-related behavior of pathogenic *V. harveyi* and attenuation of biofilm formation by the human pathogen *Candida albicans.*^46^

#### 4.1.2. Resistance to stress conditions

AI-2 also mediates biofilm formation, virulence factor expression, adhesion and colonization, and other social behaviors, thereby improving the stress resistance of the strain^47^^,^^48^ (Figure 3A). Numerous studies have reported on the regulation of biofilm formation by AI-2, such as through the exogenous addition of AI-2,^49^ and the overexpression of luxS in *Lactobacillus plantarum* L-ZS9, both of which promote bacterial biofilm formation.^50^ AI-2 was demonstrated to enhance the gastrointestinal stress resistance of *E. coli* under low-density cell culture conditions.^51^ The signal molecule AI-2 is involved in regulating biofilm formation and development and improves bile salt tolerance in *Lactobacillus sanfranciscensis.*^49^ Similarly, overexpression of AI-2E protein in *Lactobacillus acidophilus* CICC 6074 promotes AI-2 secretion and enhances survival in intestinal juice.^52^ In *Klebsiella michiganensis*, AI-2 biosynthesis and uptake were facilitated by the QS-associated *luxS* gene and *lsr* operon, resulting in improved copper toxicity resistance in mesobiotic microbial communities.^53^ Recent studies have demonstrated that collective behaviors of *E. coli* (autoaggregation and biofilm formation) are dependent on chemotaxis towards the interspecies quorum-sensing signal AI-2.^54^ And further research has found that chemotactic (*cheY*) and AI-2 signaling (*via lsrB*) promote the colonization of *E. coli* in the murine gut^55^ (Table 1).

**Table 1.** LuxS/AI-2–regulated behaviors in bacteria.

Quorum sensing phenotype	Species	Influence	Reference
Biofilm formation	*Vibrio harveyi*	Attenuated of biofilm formation by the human pathogen *Candida albicans*.	[46]
Biofilm formation	*Lactobacillus sanfranciscensis*	Enhanced bacterial cohesion and improved bile salt tolerance.	[49]
Biofilm formation	*Klebsiella michiganensis*	The mutations in the *lsr* operon, alongside the knockout of the *luxS* gene in KM strain (KMΔluxSΔlsr), significantly impaired the strain’s biofilm formation.	[53]
Biofilm formation	*Lactobacillus rhamnosus GG*	AI-2 promoted the colonization of LGG and biofilm formation to improve intestinal barrier function in an antibiotic-induced intestinal dysbiosis neonatal mouse model.	[56]
Biofilm formation	*Escherichia coli O157: H7*	Gallic acid inhibited biofilm formation by affecting flagellar assembly, chemotaxis, and expression of adhesion factor-related genes, which interfered with the transition of bacteria from reversible to irreversible adhesion.	[57]
Biofilm formation	*Escherichia coli*	*E. coli* transcription factor YncC (McbR) regulated colanic acid and biofilm formation by repressing expression of periplasmic protein YbiM (McbA).	[58]
Biofilm formation	*Escherichia coli*	AI-2 produced by *Enterococcus faecalis* promoted the autoaggregation of *Escherichia coli* and chemotaxis-dependent coaggregation between the two bacteria.	[55]
Resistance to stress	*Escherichia coli*	Enhanced the vitality of *E. coli* in simulated gastric juice.	[51]
Resistance to stress	*Lactobacillus acidophilus*	Overexpression of AI-2E protein in *Lactobacillus acidophilus* CICC 6074 enhanced their survival ability in intestinal juice.	[52]
Resistance to stress	*Klebsiella michiganensis*	*lsr* operon was involved in enhancing the organism’s tolerance to copper stress.	[53]
Resistance to stress	*Escherichia coli*	The formation of a mixed biofilm of *Escherichia coli* and *Enterococcus faecalis* enhanced the stress resistance (hydrogen peroxide) of the two strains.	[59]
Virulence factor	*Salmonella enterica Typhimurium*	AI-2 negatively controled the T3SS-1 and attenuated the virulence of the strain in infection *via* YeaJ.	[38]
Virulence factor	*Haemophilus parasuis*	ΔluxS strain attenuated its virulence about 10-folds compared with the wild-type strain.	[60]
Adhesion	*Aeromonas salmonicida*	Reduction of AI-2 production by mucins or *luxS*-deletion lead to a reduced *A. salmonicida* auto-aggregation/colonization of the gill.	[61]
Adhesion	*Escherichia coli*	Quercetin reduced the expression of genes such as adhesion, virulence, biofilm secretion, and key regulatory proteases.	[62]
Adhesion	*Escherichia coli*	AI-2 signaling (via *lsrB*) and chemotaxis (*cheY*) promoted gut colonization by *Escherichia coli*, which is in turn connected to the ability of the bacteria to utilize fructoselysine (*frl* operon)	[55]
Bacteriocin synthesis	*Lactobacillus plantanum AB*	The increase in bacteriocin significantly inhibited the *Shewanella baltica* in the shrimp samples.	[43]
Bacteriocin synthesis	*Lactobacillus reuteri NMD-86*	Remarkably increased the bacteriocin synthesis of *Lactobacillus. plantarum* NMD-17.	[63]
Spore formation	*Bacillus cereus*	Terpinen-4-ol effectively inhibited growth and biofilm and spore germination of *Bacillus cereus*.	[64]
Spore formation	*Bacillus velezensis*	AI-2 interacts with RapC to stimulate its binding to ComA, which leads to an inactive ComA and subsequently a sporulation inhibition.	[65]
Production of exopolysaccharides	*Bacillus cereus*	Terpinen-4-ol obviously reduced the strain’s extracellular matrix synthesis, especially exopolysaccharides.	[64]
Production of exopolysaccharides	*Erwinia carotovora Pseudomonas fluorescens*	Inhibition of motility, exopolysaccharide production, and biofilm formation of the strain with the decrease in the AI-2.	[66]

### 4.2. Manipulation of cell-cell interspecific interactions by AI-2

#### 4.2.1. Pathogens- pathogens

Mixed-species microecology is predominantly shaped by the interactions such as competition, cooperation and predation among community members^67^ (Figure 3B). Functioning as a universal quorum sensing signal, AI-2 is synthesized by over 50% of sequenced bacterial species, exhibiting significantly broader interspecies communication capacity compared to AI-1.^68^ Existing investigations have increasingly focused on AI-2-mediated interactions among defined symbiotic bacterial pairs. Symbiotic bacteria engage in competition for space by rapidly colonizing uninhabited niches or competing with already established populations.^69^ AI-2 produced by *Clostridium difficile* affects the formation of multispecies biofilms of *Bacteroides fragile.*^70^ Conversely, *Bacteroides fragilis* inhibits the growth, biofilm formation, and virulence of *Clostridium difficile* by inducing a metabolic response.^70^ AI-2 production by *Streptococcus gordonii* mediates dual-species biofilm formation with *Porphyromonas gingivalis* through regulation of carbohydrate metabolism.^71^ In the co-culture system, AI-2 molecules play a crucial role in the physical interaction (the exchange of cytoplasmic molecule) between *Desulfovibrio vulgaris* Hildenborough (*D. virgaris*) and *E. coli*, thereby affecting the metabolic activity of *D. Virgaris.*^72^

#### 4.2.2. Pathogens- probiotics

Additionally, AI-2, which has been proposed to serve as a universal language, plays an essential role in mediating interactions between probiotics and pathogens.^73^ The production of adhesins and receptors that bind to specific surface features provides a competitive advantage for colonizing unoccupied niches and prevents displacement by invaders.^74^ Similar quorum quenching phenomena have been observed in various pathogenic bacteria and probiotics. High performance liquid chromatography analysis revealed that the presence of *luxS* in *Vibrio vulnificus* within the gut impacts the sensitivity of its symbiotic bacteria (such as *Lactobacillus*) to growth-inhibiting activity.^75^ *Lactobacillus sakei NR288* significantly decreased cell viability, AI-2 activity, and expression of virulence factors associated with enterohaemorrhagic *E. coli* O157: H7.^76^ *Lactobacillus* adhering to human epithelial cells promotes colonization of the strain and defends against pathogen attachment by producing extracellular glycoproteins.^69^ Some strains of *Lactobacillus* and *Bifidobacterium* have been evaluated for their efficacy in inhibiting the growth, viability, biofilm formation, and co-aggregation of *Aggregatibacter actinomycetemcomitans*, *Streptococcus mitis* and *Streptococcus mutans.*^77^ The AI-2/LuxS system modulates the nutritional competitiveness of *Lactobacillus plantarum* SS-128 by facilitating balanced energy expenditure and enhancing membrane transport systems, thereby inhibiting the growth of *Shewanella baltica.*^78^

#### 4.2.3. Probiotics- probiotics

AI-2 also mediates interspecific interactions among lactic acid bacteria.^79‐81^ The AI-2/LuxS system regulates the cooperation between *Lactobacillus plantarum* AB-1 and *Lactobacillus casei*, and co-culture significantly enhances the antibacterial activity and transcription of the QS *luxS* gene and bacteriocin regulatory operons (plnB and plnC) in *Lactobacillus plantarum* AB-1.^43^ Co-cultivation increases the activity of *Lactobacillus plantarum* NUC08 AI-2, promoting synergistic effects with yogurt fermentation strains and improving the growth of mixed strains and their tolerance to simulated gastric and intestinal conditions.^82^ Using gene knockout technology, it was shown that the LuxS/AI-2-mediated QS system plays a crucial role in bacteriocin synthesis of *Lactobacillus plantarum* NMD-17 in co-culture.^63^ Notably, bacteriocin synthesis in *Lactobacillus plantarum* NMD-17 is markedly induced during co-cultivation with *Lactobacillus reuteri* NMD-86 with increases in cell numbers and AI-2 activity. The expression of *luxS*, which encodes the AI-2 synthetase, and *plnB*, *plnD*, *plnE*, and *plnF*, which encode components of the bacteriocin system, is significantly upregulated in co-cultivation.^63^ In contrast, the yeast-lactic acid bacterium (LAB) co-culture system has been less studied. Previous research has reported AI-2-regulated signal transduction between *Lactobacillus paracasei* B1 and *Pichia* sp. J1, mediating cooperative behavior between yeast and LAB.^83^

### 4.3. Manipulation of multi-species interspecific interaction by AI-2

#### 4.3.1. Synthetic microbial community

The scope of AI-2-mediated quorum sensing research has evolved from dual-strain to complex multispecies systems.^84^ As shown in Figure 3C, Novel synthetic microbial community models have been established in recent years to elucidate QS regulatory mechanisms during ecological succession.^85‐89^ AI-2 transporters have been identified as critical mediators of interspecies crosstalk, maintaining community equilibrium in model consortia containing *Pseudomonas aeruginosa*, *Pseudomonas protegens*, and *Klebsiella pneumoniaee.*^67^ Community restructuring was demonstrated to arise from synergistic pairwise interactions, where AI-2 transporters optimize collective biomass production.^67^ Within oral biofilm microbiota, AI-2 signaling ablation was shown to delay biofilm matrix polymerization, compromise structural integrity, and prolong colonization by both pioneer (*Streptococcus* spp.) and late-stage taxa (*Prevotella*, *Clostridium*).^90^ In an *in vitro* co-culture system, *Bifidobacterium longum*, *Bacteroides ovatus*, *Enterococcus faecalis*, *and Lactobacillus gasseri* exhibit synergistic interactions, with enhanced biofilm formation capabilities.^91^ As the biofilm architect, *Bifidobacterium longum* upregulated the metabolism of autoinducer peptides (proliylglycine and glycylleucine), *N*-acyl homoserine lactone (*N*-(3-oxo hydroxy) homoserine lactone), and AI-2.^91^ Additionally, the *luxS* gene was further demonstrated to potentiate iron scavenging in *Bifidobacterium.*^92^ This competition for ecological niche, driven by iron clearance, plays a vital role in preventing infections caused by entero-hemorrhagic *E. coli* and *Citrobacter rodentium.*^93^^,^^94^

#### 4.3.2. Gut microbiota

The gastrointestinal tract harbors a complex microbial ecosystem characterized by intricate bacterium-bacterium and host-microbe interactions^95^ (Figure 3C). Within this multispecies consortium, intercellular communication is mediated by chemical signaling molecules including quorum sensing systems, enabling environmental sensing and adaptive responses.^96^ AI-2 QS has been widely implicated in microbial colonization dynamics and chemotactic behavior within the intestinal niche.^96^ Comparative analysis of wild-type versus *ΔluxS Vibrio* strains revealed significantly enhanced jejunal and ileal colonization by *V*i*brio vulnificus* in the presence of intact AI-2 signaling.^75^ Similarly, Exogenous AI-2 supplementation was shown to potentiate *Lactobacillus rhamnosus* colonization in antibiotic-perturbed murine intestines.^56^ QS activation involves bidirectional crosstalk, where bacterial populations both perceive environmental cues and reciprocally modulate their ecological niche. AI-2 concentration gradients have been demonstrated to shape community structure within the gut ecosystem.^97^ For instance, in *E. coli*-derived AI-2 elevates the Firmicutes/Bacteroidetes ratio, thereby counteracting antibiotic-induced dysbiosis.^98^ AI-2-associated genomic plasticity in *E. coli* has been proposed to facilitate niche partitioning and stable cohabitation in intestinal ecosystems.^98^ Another study has found that mammalian epithelial cells produce AI-2 mimic in response to bacteria or tight-junction disruption, indicating that the AI-2 QS system might play a key role in promoting cross-kingdom signaling.^99^

#### 4.3.3. Natural ecology

Natural microbial consortia exhibit remarkable complexity, where symbiotic and competitive interactions are governed by quorum sensing mechanisms^9^ (Figure 3C). AI-2-mediated signaling has been implicated in symbiotic homeostasis maintenance between microbiota and their hosts.^100^^,^^101^ In nature, the surface mucus layer of corals harbors an abundance of QS bacteria, and the function of QS regulation has a significant impact on the health of corals.^101^ For instance, in natural reef-building corals, AI-2 significantly contributed significantly to an increase in coral bleaching, altered the ratio of potential probiotic and pathogenic bacteria, and suppressed the antiviral activity of specific pathogenic bacteria while enhancing their functional potential, such as energy metabolism, chemotaxis, biofilm formation and virulence release.^100^ Genomic and metatranscriptomic profiling of rumen microbiota identified eight distinct QS signal classes, confirming AI-2’s pivotal role in cross-species communication within this specialized niche.^37^^,^^102^ AI-2 recognition by dCache_1 domains was demonstrated to activate CahRs, thereby orchestrating collective microbial behaviors. The rumen ecosystem is essential for lignocellulose degradation, nutrient assimilation, and host energy provision.^37^

## 5. Application of AI-2 mediated interspecies communication

The AI-2 QS system serves as a critical mediator of interkingdom signaling with multifaceted applications. In addition to examining the role of AI-2 mediated interactions in host health, this paper systematically explores the emerging applications of AI-2 across various fields, including agriculture, industry, and environmental ecology.^47^

### 5.1. Host-microbe engineering: maintain human health

#### 5.1.1. Regulation of the intestinal microbiome

**Figure 4.**
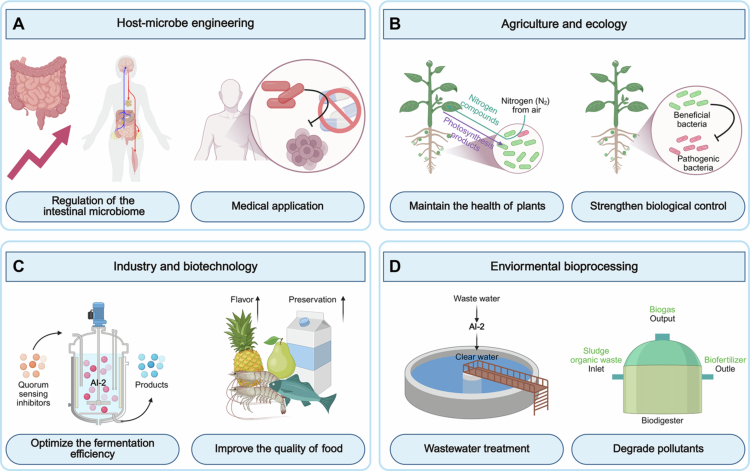
The application of AI-2-mediated interspecific communication across four major fields. (A) Host-microbe engineering. AI-2-based strategies are used to modulate host-microbe interactions, such as microbiota remodeling and medical applications, to improve human health. (B) Agriculture and ecology. AI-2 is employed to regulate plant-microbe symbioses, control agricultural pathogens, and enhance ecological stability in agricultural ecosystems. (C) Industry and biotechnology. AI-2-mediated quorum sensing is harnessed to optimize industrial fermentation processes, to improve the production of bioproducts and to enhance the quality of food. (D) Environmental bioprocessing. AI-2-based approaches facilitate pollutant degradation, heavy metal bioremediation, and the regulation of microbial communities in environmental restoration. AI-2-mediated interspecific communication has broad translational potential, offering innovative solutions for challenges in health, agriculture, industry, and environmental sustainability.

The AI-2 QS system is prevalent within the intestinal microbiota^37^^,^^97^ (Figure 4A). Under ecological imbalance conditions caused by antibiotics or inflammation, the chemotaxis of AI-2 can lead to the microbiota suppression of the *Enterobacteriaceae* microbiota.^55^^,^^103^^,^^104^ AI-2 signals are known to influence the composition, stability, and interactions of the intestinal microbiota with the host. Probiotics are employed to utilize AI-2 for regulating their own behaviors and interactions with both the host and other bacteria, which may potentially enhance their colonization efficiency and functional capabilities. Concurrently, they are also capable of disrupting the colonization processes and virulence expression of intestinal pathogens, such as pathogenic *E. coli* and *Salmonella*. Therefore, AI-2 may maintain barrier integrity and reduce inflammation through the regulation of the microbiota.^56^ For instance, an analysis of population and animal experimental data has revealed that AI-2 concentrations significantly decrease during the acute phase of necrotizing enterocolitis (NEC) and gradually increase during recovery.^105^ Thus, AI-2 levels are correlated with different disease stages, suggesting its potential as a novel biomarker for diagnosing and monitoring NEC.^105^ In antibiotic-treated mice, AI-2 supplementation (10 μM) has been shown to restore *Lactobacillus rhamnosus* colonization, thereby reducing intestinal inflammation by 60%.^56^Also, the homeostasis of the intestinal microbiome is also known to affect the health status of other organs in the host body. An analysis of AI-2 levels and gut microbiome composition in stools from pneumonia patients and in a mouse model of acute lung injury has shown that gut microbiome-derived AI-2 can modulate lung inflammation *via* the entero-pulmonary axis.^106^

#### 5.1.2. Medical applications - anti-infection strategies

In contrast to traditional antibiotics, anti-QS therapy that targets AI-2 was shown to mitigate the development of drug resistance.^46^^,^^107^ AI-2 produced by bacteria or artificially synthesized AI-2 analogs can bind to receptors without activating signaling pathways, thereby blocking AI-2 signaling. For instance, CT-26 tumor growth was suppressed by 75% in murine models by engineered *Salmonella* expressing AI-2-responsive cytolysin A (ClyA), thereby highlighting AI-2’s therapeutic potential.^108^ The adhesion of *Pseudomonas aeruginosa* biofilms to respiratory epithelial cells was reduced by AI-2 analog (D-ribose),^109^^,^^110^ presenting a novel therapeutic strategy for patients with cystic fibrosis.^110^ Additionally, the use of QS quenching technology (utilizing enzymes or functional bacteria to break down signaling molecules used for communication between microbes) was shown to reduce environmental AI-2 concentrations, thereby achieving the same goal of interfering with signal transduction.^111‐113^ AI-2 QS inhibitors were analyzed and screened through multiple molecular techniques in a recent study, targeting 5’-methylthioadenosine/S-adenosylhomocysteine nucleosidase (MTAN) to combat drug-resistant *Helicobacter pylori.*^114^ Another study has found that furanones inhibit signaling by covalently modifying and inactivating the AI-2 producing enzyme LuxS.^9^^,^^115^

### 5.2. Agriculture and ecology: optimization of plant-microbial interaction

#### 5.2.1. Maintain the health of plants

The rhizosphere of plants is also a complex microbial community environment, in which the abundant AI-2 mediates the interactions among rhizosphere bacteria^116^ (Figure 4B). The collaboration and stability of beneficial microbial communities may be enhanced by inoculating AI-2-producing strains such as plant growth-promoting rhizobacteria (PGPRs).^117^ Additionally, it promotes plant growth and induces systemic resistance.^118^^,^^119^ Qin inoculated the beneficial plant bacterium *Bacillus velezensis* SQR9, which was isolated from the rhizosphere of cucumber plants, into the maize seedling rhizosphere.^120^ The results indicated that AI-2 enhanced the viability of the strain, biofilm formation, and root colonization.^120^ The latest research has found that the inclusion of two crucial collaborators (*Lysobacter* and *Microbacterium*) could efficiently foster the colonization of PGPR and aid PGPR in executing phytoremediation enhancement.^121^

#### 5.2.2. Strengthen biological control

The reliance on chemical pesticides and fertilizers in modern crop production has posed significant challenges for the environment and ecology^122^^,^^123^ (Figure 4B). There is a growing need to develop more environmentally friendly alternatives to promote the development of sustainable agriculture.^124^ The AI-2 signal has been utilized to coordinate the introduction of biological control bacteria, enhancing their colonization in the rhizosphere, biofilm formation, and production of antibacterial substances. This approach thereby strengthens their inhibitory effect on plant pathogenic bacteria such as *Agrobacterium* and *Ralstonia.*^125^ *Bacillus* strains, serving as biological control agents against plant pathogenic bacteria, have demonstrated the potential to control various plant pathogenic bacteria and inhibit quorum induction.^126^ The plant growth-promoting rhizobacterium *Acidovorax radices* N35 has been shown to alleviate the earthworm-mediated increase in pest abundance, particularly in the ambient environment.^124^

### 5.3. Industry and biotechnology: regulation of microbial fermentation

#### 5.3.1. Optimize the fermentation efficiency of the mixed bacterial community

Owing to its high economy and high efficiency, microbial fermentation has been widely applied in industrial production (Figure 4C). In complex fermentation systems, AI-2 has been shown to be involved in a wide range of industrial production applications. During bioethanol production, supplementation with AI-2 quorum sensing inhibitors (0.2 g/L DMHF [2,5-dimethyl-4-hydroxy-3(2 H)-furanone] or 18.0 g/L D-galactosamine) reduced AI-2 activity by 38% and 36%, respectively.^127^ This intervention not only inhibited biofilm formation by contaminating *Lactobacillus plantarum* strains but also enhanced ethanol yields by 14% and 103%.^127^ Similarly, in anaerobic membrane bioreactors (AnMBRs), 10 μM AI-2 supplementation increased methane production by 38% through upregulation of *mcrA* gene expression in methanogenic archaea.^128^ Furthermore, metabolic profiling revealed that AI-2 induction shifts *Zymomonas mobilis* metabolism toward ethanol biosynthesis, suggesting potential applications in second-generation ethanol production.^129^

#### 5.3.2. Improve the fermentation flavor and preservation of food

AI-2 has been implicated in regulating metabolic pathways that can influence food flavor^130^ (Figure 4C). 600 nmol/L DPD can enhance the flavor formation of *Lactobacillus plantarum* HRB1 and significantly increase the content of free amino acids, aldehydes, ketones, alcohols and other substances in fermented dry sausages.^131^ *Lactobacillus fermentum* A119 mediated the expression of aldehyde dehydrogenase, converting benzaldehyde (a metabolite of beany flavor) into benzoic acid, thereby improving the flavor of soy protein-based fermented milk.^132^

In addition to its influence on the flavor of fermentation, the interspecific interaction mediated by AI-2 can also be used for the preservation of food. The addition of *Lactobacillus plantarum* SS-128 (serving as a biocontrol bacterium) effectively slowed down protein degradation by inhibiting the growth of food pathogens, and the increase in total volatile basic nitrogen value was relatively slow.^133^ Further research found that exogenous 25 mM L-cysteine can significantly enhance the AI-2/LuxS system of *Lactobacillus plantarum* SS-128, regulate interspecific communication within biofilms, and significantly inhibit the growth of *Vibrio parahaemolyticus* and *Shewanella putrefaciens* grown on shrimp and squid surfaces.^42^ The addition of AI-2 increased the abundance of *Bacillus amyloliquefaciens* and enhanced the biological control effect on *Penicillium* in pears and loquats by stimulating the growth of *Bacillus amyloliquefaciens* and inducing the formation of biofilms.^107^

### 5.4. Environmental bioprocessing: pollutant degradation and ecological restoration

#### 5.4.1. Microbial community regulation in wastewater and sludge treatment

Emerging evidence suggests that the AI-2-mediated QS system indirectly enhances the treatment efficiency of wastewater and sludge by regulating the composition and individual metabolism in the microbial community (Figure 4D). AI-2-mediated interspecific communication plays a pivotal role in wastewater and sludge treatment dynamics.^134^ In AnMBRs, facultative quorum quenching consortia targeting AI-2 signaling enhance biofouling control by 35-40% through QS disruption.^27^^,^^135^^,^^136^ On the one hand, AI-2 is extensively present in anaerobic wastewater treatment and has great potential for accelerating anaerobic granular sludge formation, promoting system stability, and boosting organic matter degradation.^128^^,^^135^ AI-2-mediated QS can improve anaerobic wastewater treatment by regulating amino acid synthesis and gluconeogenesis, thereby affecting extracellular polymeric substance (EPS) production. It also promotes hydrolysis, acidification, and the synthesis of key enzymes for methanogenesis, as well as regulates the synthesis of electron carriers to facilitate interspecies electron transfer.^128^ On the other hand, AI-2 has been applied in anti-fouling strategies for wastewater treatment systems.^128^ The addition of *Acinetobacter* sp. DKY-1-entrapping beads to a membrane bioreactor significantly decreased DPD concentration and remarkably reduced membrane biofouling.^137^ Subsequent studies found that the newly isolated strain *Pantoea* sp. PL-1 demonstrated superior performance compared to the DKY-1 strain in minimizing the impact of membrane bioreactor fouling, with a 40% improvement in performance.^138^ Also, similar reactions occur in sludge treatment.^139^ or instance, AI-2-mediated QS can partially regulate the toxic shock response of anaerobic sludge by regulating the activities of thick-walled and cotrophic bacteria, thereby providing a new method for shortening the recovery time of the anaerobic process.^140^

#### 5.4.2. Environmental residual pollutant removal

Additionally, AI-2-mediated interspecific communication can be applied to remove residual pollutants in the environment, such as herbicides and hydrogen peroxide (Figure 4D). Atrazine, a common triazine herbicide, has posed a threat to ecological security due to its high water solubility.^141^ The biodegradation rate of atrazine has been significantly enhanced by *Paenarthrobacter* sp. KN0901 when combined with phosphorus-doped hydrochar through the regulation of AI-2.^142^ Hydrogen peroxide is widely used to treat bacterial and parasitic infections. AI-2 provides a safe, efficient, and low-cost mechanism for hydrogen peroxide removal, thereby preventing severe damage to aquatic animals caused by overuse.^72^^,^^143^ AI-2 contributes to the resistance of *Deinococcus* sp. Y35 to oxidative stress induced by hydrogen peroxide by altering the membrane permeability of strain Y35, allowing more hydrogen peroxide to enter and be degraded within the bacterial cells.^144^ Furthermore, research reports that curcumin and 10-undecenoic acid, as natural QS inhibitors of *Bacillus subtilis luxS*/AI-2, can serve as alternatives to counteract bacterial pathogenicity and virulence, thereby avoiding the selection pressure typically associated with classic industrial disinfection and antibiotic treatments.^145^

## 6. Regulation of AI-2 production by various factors

**Figure 5.**
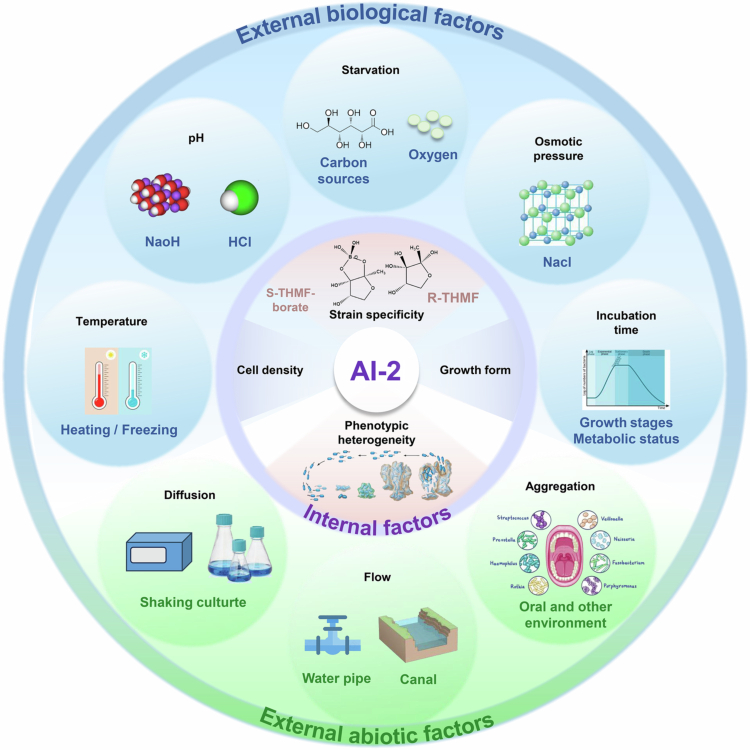
Summary of internal and external factors (biological and abiotic) affecting AI-2 production in bacteria. Internal factors include strain specificity, cell density, growth form, and phenotypic heterogeneity. External factors comprise two subcategories: (1) Biological factors (such as nutritional status and environmental stress); (2) Abiotic factors (such as diffusion, flow, and aggregation). This figure provides a framework for understanding the context-dependent nature of AI-2 signaling in complex environments and for designing AI-2-informed control strategies.

The regulation of AI-2 in different bacterial environments is extremely complex (Figure 5). Internally, bacterial growth density and growth state (planktonic or biofilm) significantly impact AI-2 levels and QS responses, with phenotypic heterogeneity observed in QS-related gene expression. Externally, biological factors such as nutritional status and environmental stress, as well as abiotic factors like aggregation, diffusion, and flow, also influence AI-2 synthesis and QS.

### 6.1. Regulation of AI-2 production by internal factors

#### 6.1.1. Influence of growth density

The growth stage and cell density of bacteria influence the production, accumulation, and QS response of AI-2. QS refers to the collective behavior that occurs when extracellular signaling molecules reach a certain threshold. Initial research on QS revealed that cells exhibit varying degrees of QS at low cell density and high cell density, with the assumption that the QS phenotype remains consistent at a given concentration. However, transient heterogeneity exists even among individual bacteria at the same concentration. For instance, AI-2 regulates bioluminescence in *V. harveyi*, and while most cells luminesce at high cell density, a small fraction remains dark.^146^ Similar heterogeneity is observed in the AHLs QS system. In *Pseudomonas putida*, AHLs regulates some bacteria for motility and others for biofilm formation at low density, but at high density, all cells synchronize their commitment to motility.^147^ Furthermore, research has shown that heterogeneity persists in QS responses even at high cell density and with adequate AHLs supplementation from external sources.^148^

#### 6.1.2. Differences in cell growth states

Bacteria can exist as planktonic cells or biofilm cells, and their growth form influences QS responses.^149^ Biofilms, a self-protective adaptation to resist adverse environments, are the predominant form of bacteria in nature.^150^ Compared to planktonic cells, biofilm cells have a more active pathway for AI-2 synthesis and metabolism. During the biofilm’s maturation stage, bacterial metabolic waste accumulates in limited spaces through gap water channels, leading to intra- or inter-species competition.^49^ In this context, the AI-2-mediated QS system regulates the detachment of some bacteria from the biofilm, resulting in the formation of planktonic cells in a dispersed phase.^151^^,^^152^ This cycle helps limit the accumulation of metabolic waste, nutrient deficiencies, and other unfavorable conditions for bacterial growth.^153^^,^^154^ For probiotics, biofilm formation enhances tolerance to the gastrointestinal environment, significantly impacting the regulation of intestinal microbial diversity.^155^^,^^156^ Additionally, biofilm probiotics exhibit superior immune regulation compared to their planktonic counterparts.^157^ For pathogenic bacteria, biofilm formation hinders the spread of antibacterial agents, increasing drug resistance and making it more challenging to eliminate or reduce infections.^158^ Besides, single-cell RNA-seq analyzes reveal that AI-2 signaling induces phenotypic heterogeneity within biofilms, where subpopulations exhibit divergent metabolic states.^159^^,^^160^ For example, in *E. coli* biofilms, AI-2-high cells upregulate *lsr* operon expression, while AI-2-low cells prioritize motility genes.^161^ This spatial division enhances community resilience to antibiotics.^7^^,^^55^^,^^158^

#### 6.1.3. Exploration on heterogeneity

Historically, it was often assumed that genetically identical bacteria exhibit similar behaviors under the same environmental conditions, leading to the initial assumption of homogeneity in the concept of QS.^54^^,^^55^ Essentially, QS is a synchronized behavior where all group members participate uniformly in the production of autoinducers and the activation of target genes. However, over the past decade, advancements in single-cell analysis techniques have revealed phenotypic heterogeneity in the expression of QS-related genes in many bacterial species. This heterogeneity is evident in both autoinducer production and target gene activation levels.^159^ In other words, some cells may not participate in the coordinated behavior but still benefit from the collective cooperation. Spatial disorder, a common phenomenon observed in bacterial colonies, significantly influences colony growth and induction dynamics, contributing to the heterogeneity of QS responses.^162^ This spatial heterogeneity can lead to high local cell densities where QS may initiate before the entire community is induced.^162^ Despite this, reports on QS heterogeneity are limited, and its sources are likely multifactorial.^159^ It has been speculated that the adhesion properties of biofilms provide a platform for phenotypic heterogeneity to adapt to host niches.^163^ The causes of heterogeneity remain unclear, and many questions remain unanswered.^159^ For instance, how prevalent is QS heterogeneity? What are the advantages and disadvantages of this phenomenon for collective cooperation? And can heterogeneity be inherited?

### 6.2. Regulation of AI-2 synthesis by external conditions

#### 6.2.1. External biological factors

Aside from the specificity of bacterial strains, biological factors- including nutritional status and stress in the external environment- can also influence the production and regulation of AI-2.^65^^,^^164^ Bacteria’s ability to precisely sense alterations in their in their local environments and monitor gradients of nutrients, chemical signals, and oxygen concentration is crucial for gene-regulation-based adaptation mechanisms. These mechanisms ultimately provide evolutionary advantages for the growth and survival strategies of certain bacterial strains or colonies.^162^ Dina Ramic developed a whole-cell biosensor assay to determine that AI-2 production in *Campylobacter jejuni* 81-176 is dependent on the growth medium.^165^ Compared to the HPLC-FLD method, this biosensor offers a lower detection limit, which helps clarify the linear relationship between AI-2 production and cell density in this strain.^165^ Research on AnMBRs used for treating low-intensity wastewater, including urban sewage, has shown that starvation can lead to increased AI-2 levels.^134^ Moreover, biopolymers, biosolids, volatile fatty acids, and alkalinity levels are positively correlated with AI-2 concentrations.^134^ Similar studies have shown that starvation induces AI-2 accumulation in anaerobic bioreactors, correlating with enhanced extracellular polymeric substance (EPS) synthesis (R² = 0.87).^134^ Stress responses to factors such as pH, bile acids, temperature, osmotic pressure, and starvation induce species- and strain-specific regulation of AI-2 activity in *Lactobacillus rhamnosus* and *Lactobacillus plantarum.*^166^^,^^167^ For instance, acid stress enhances the QS response in *Lactobacillus*, as evidenced by increased transcription of *luxS* genes and AI-2 activity.^168^ In response to acid stress, *Bifidobacterium longum* regulates biofilm formation through the AI-2/QS, cAMP, and LuxC/LuxE TCS systems by modulating Tad IV pilin and extracellular substance secretion.^169^ Temperature also affects QS, with the coral pathogen *Vibrio couliilyticus* increasing the expression of several QS proteins and AI-2 production in response to higher ambient temperatures.^170^ Further information regarding stress conditions and the production of AI-2 is provided in Table 2.

**Table 2.** Synthesis of AI-2 induced under extreme conditions.

Classification	Strain	Origin	Quorum sensing inhibitors	High temperature	Acid	Starvation	Osmotic pressure	Reference
Probiotic	*Lactiplantibacillus plantarum ATCC8014*	Chinese sauerkraut	DMHF; D-Gal	ND	ND	ND	ND	[127]
	*Lactobacillus fermentum* 2-1	Horse milk	ND	++	+++	+	−	[166]
	*Lactobacillus rhamnosus* GG	Maasai milk	ND	++	++	–	+	]^167^]
	*L. rhamnosus* BFE5264	Kimchi	ND	++	++	−	+	[167]
	*L. plantarum* 299v	Kimchi	ND	++	+	+++	+	167
	*L. plantarum* NR74	Kimchi	ND	−	NS	+	+	[167]
	*Lactobacillus rhamnosus* GG	Human feces	ND	ND	+	ND	ND	[168]
	*Lactobacillus acidophilus* NCFM	Human feces	ND	ND	+	ND	ND	[168]
	*Bifidobacterium longum* FGSZY16M3; FSHHK13M1; FSHHK22M1; FJSWXJ10M2	Human feces	ND	ND	+	ND	ND	[169]
	*Lactobacillus*	Food waste	Oleic acid; stearic acid	ND	ND	ND	ND	[171]
Pathogenic bacteria	*Cronobacter malonaticus* G362	ND	ND	ND	ND	ND	+	[172]
	*Vibrio couliilyticus* ATCC BAA-450	Diseased coral of the species *Pocillopora damicornis*	ND	+	ND	ND	ND	[170,173]
	*Klebsiella*	Food waste	Oleic acid; stearic acid	ND	ND	ND	ND	171
	*Streptococcus suis* HA9801	Diseased swine	Paeoniflorin	ND	ND	ND	ND	[174]
	*Bacillus cereus* ATCC 10987; ATCC 14579	Corrupt cheese	Camellia saponins	ND	ND	ND	ND	[175]
	*Campyloba jejuni* F38011; NCTC11168	Human clinical isolate	Decanoic acid; lauric acid	ND	ND	ND	ND	[176]
	*Escherichia coli O157: H7*	Human feces	ND	ND	+	ND	+ + +	[177]

#### 6.2.2. External abiotic factors

In addition to biological factors in the external environment, abiotic factors such as aggregation, diffusion, and flow also influence the production and regulation of QS signals.^164^ Research by Minyoung Kevin Kim suggests that the dynamics of QS under varying flow conditions arise from complex interactions among bacterial signaling pathways, the diffusion of small molecules, convection, biofilm structure and thickness, diffusion pathways within cellular aggregates, as well as the distances and configurations of flow networks.^178^ For instance, fluid shear stress (5 mL/min) disrupts AI-2 gradients in Pseudomonas aeruginosa biofilms, delaying QS activation.^178^ Most distinct molecular classes identified in the *Pseudomonas* metabolome under both shaking and static conditions are associated with QS and signaling compounds.^179^ By simulating the structured surface of the mammalian gut, it was found that in the presence of flow, the physical structure of the environment affects AI-2 gradients, promoting the chemotactic accumulation of *E. coli* in Dead-End Pores (DEPs).^180^ Apart from flow factors, differences in surface topography can also cause temporal and spatial inhomogeneity of autoinducing peptides (AIPs).^178^ Although there are fewer similar studies on AI-2, it is generally accepted that the amount of bacterial biomass required to initiate QS in a particular bacterial population increases with higher fluid flow rates.^4^

## 7. Future perspectives

Due to limitations in scientific and technological capabilities, previous knowledge of AI-2’s interspecific interactions primarily focused on the collective level. However, with the advancement of single-cell sequencing technology, it has been reported that QS enhances the overall adaptability of bacterial communities by both coordinating group behaviors and influencing individual characteristics.^160^^,^^161^ And the specific variations in individual cell responses during interspecific interactions have not been thoroughly investigated.

Furthermore, current research often focuses on a single QS effect within a system, leaving the coordination mechanisms of multiple and complex QS systems in interspecific interactions within microecology poorly understood. In the same biological environment, there may be antagonistic interactions between multiple QS systems.^181^ For instance, interactions between multiple strains often involve multi-type/multi-unit QS systems, including intraspecific and interspecific communication *via* AI-2/AHLs and intraspecific communication *via* AIP.^182^^,^^183^ Such microbiota could use two or more QS systems to encode hierarchical activation of QS.

In addition, there are still several emerging frontiers to explore: 1) AI-2 signaling in non-bacterial kingdoms (for instance, fungal AI-2 mimicry^173^); 2) Metabolite-QS crosstalk (for instance, synergistic/antagonistic effects of short-chain fatty acids on AI-2 signaling^171^); and 3) AI-2-based “smart” biofilms for real-time environmental monitoring.^127^ Addressing these challenges necessitates the utilization of interdisciplinary tools, ranging from single-cell metabolomics to the design of synthetic consortia. Such interdisciplinary approaches will be crucial for fully leveraging the potential of AI-2 in microbial communication and for advancing biotechnological applications.

## 8. Conclusions

AI-2 serves as a universal signaling molecule governing microbial interactions, metabolic coordination, and ecological resilience. This review highlights AI-2’s dual structural adaptability (S-THMF-borate/R-THMF) and its hierarchical integration with QS systems (such as AHLs) to mediate cross-species communication. Diverse receptors (LuxP, LsrB, etc.) underpin its species-specific perception, though Gram-positive mechanisms remain underexplored. AI-2 dynamically regulates metabolic flux, biofilm formation, and multi-species interactions in synthetic consortia, gut microbiota, and environmental systems. AI-2-mediated interspecific communication has been extensively utilized across various domains, including host health, agriculture, industry, and environmental ecology. The production of AI-2 is modulated by growth phases, environmental stressors, and physicochemical factors. Future priorities include resolving phenotypic heterogeneity in QS responses, decoding AI-2’s role in non-bacterial domains, and integrating multi-QS crosstalk for synthetic ecology applications. Bridging molecular insights with engineering frameworks will unlock AI-2’s full potential in microbial resource utilization.
